# A Comparative Study on the Multiscale Mechanical Responses of Human Femoral Neck Between the Young and the Elderly Using Finite Element Method

**DOI:** 10.3389/fbioe.2022.893337

**Published:** 2022-05-05

**Authors:** Haipeng Cen, He Gong, Haibo Liu, Shaowei Jia, Xiaodan Wu, Yubo Fan

**Affiliations:** ^1^ Key Laboratory for Biomechanics and Mechanobiology of Ministry of Education, Beijing Advanced Innovation Center for Biomedical Engineering, School of Biological Science and Medical Engineering, Beihang University, Beijing, China; ^2^ School of Engineering Medicine, Beihang University, Beijing, China

**Keywords:** aging, femoral neck, multiscale finite element models, biomechanical responses, cortical bone, osteocyte lacuna-canalicular network and extracellular matrix (OLCEM)

## Abstract

**Background:** Femoral neck fracture (FNF) is the most serious bone disease in the elderly population. The multiscale mechanical response is a key to predicting the strength of the femoral neck, assessing the risk of FNF, and exploring the role of mechanosensation and mechanotransmission in bone remodeling, especially in the context of aging bone.

**Methods:** Multiscale finite element (FE) models of the proximal femur for both young and elderly people were developed. The models included organ scale (proximal femur), tissue scale (cortical bone), tissue element scale (osteon), and cell scale [osteocyte lacuna-canalicular network (LCN) and extracellular matrix (ECM), OLCEM]. The mechanical responses of cortical bone and osteocytes in the mid-femoral neck and the differences in mechanical responses between these two scales were investigated.

**Results:** The mechanical responses of cortical bone and osteocyte showed significant differences between the elderly and the young. The minimum principal strains and mean SEDs of cortical bone in the elderly were 2.067–4.708 times and 3.093–14.385 times of the values in the young, respectively; the minimum principal strains and mean SEDs of osteocyte in the elderly were 1.497–3.246 times and 3.044–12 times of the values in the young, respectively; the amplification factors of minimum principal strain in the inferior (Inf), anterior (Ant), and posterior (Post) quadrants in the young were 1.241–1.804 times of the values in the elderly, but the amplification factor of minimum principal strain in the superior (Sup) quadrant was 87.4% of the value in the elderly; the amplification factors of mean SED in the young were 1.124–9.637 times of the values in the elderly.

**Conclusion:** The mass and bone mineral density (BMD) of cortical bone in the femoral neck is closely related to the mechanical response of osteocytes, which provides a new idea for improving cortical bone quality. Perhaps cortical bone quality could be improved by stimulating osteocytes. Quadrantal differences of bone quality in the mid-femoral neck should be considered to improve fracture risk prediction in the future.

## Introduction

Femoral neck fracture (FNF) is the most serious bone disease in the elderly due to its high morbidity and mortality, which is a serious threat to the health of the elderly and causes a huge economic burden for patients (Chen et al., 2014; Veronese and Maggi, 2018; Guzon-Illescas et al., 2019). FNF is closely related to the biomechanical response of femoral neck and occurs when the load on the femoral neck exceeds its strength. Therefore, biomechanical characteristics and responses of the femoral neck have always been the focus of research.

Based on engineering principles, the strength of bone depends on the material properties, the morphology, and the loading conditions in terms of magnitude, rate, and direction of applied force (Hart et al., 2017). FNF is believed to be closely related to the geometry of the femur, which has been verified by investigating the geometric parameters of the proximal femur and the recorded incidence of FNF (Faulkner et al., 1993). Subsequently, it was found that bone mineral density (BMD) of the femoral neck measured by dual-energy X-ray absorptiometry (DXA) could evaluate bone quality and predict FNF very well (Rivadeneira et al., 2007). The biomechanical response of femoral neck is to some extent dependent on its geometric shape and BMD, which has been verified in the literature (Carpenter et al., 2011). Previously, strain gauges have been used to measure the surface strain of femoral neck (Cristofolini et al., 2009). However, only the surface strain of femoral neck can be measured at limited locations. With the rapid development of computer technology, finite element method (FEM) has been widely used to study biomechanical responses. FEM can overcome this limitation effectively. The mechanical response of femoral neck was investigated using FEM, and the stress and strain distributions at any position of the femoral neck could be acquired (Kersh et al., 2018).

The aforementioned studies mainly focused on mechanical response analyses at the tissue scale. However, bone has a complex hierarchical structure, including organ scale (whole bone), tissue scale (cortical and cancellous bones), tissue element scale (osteon), and cell scale [osteocyte lacuna-canalicular network (LCN) and extracellular matrix (ECM), OLCEM] (Reznikov et al., 2014). Recently, multiscale FE method has been used to investigate the mechanical responses of bone. From the perspective of biomechanics, multiscale FE models of bone were developed to investigate the mechanical behavior of bone, e.g., organ and tissue two scales FE models of bone [radius and cortical bone (Johnson and Troy, 2017)] and organ, tissue and tissue element three scales FE models of bone [proximal femur, cortical bone and osteon (Ascenzi et al., 2013)]. However, the cell scale is not considered. In fact, the mechanical responses at the cell scale were equally important, as the mechanical responses of osteocytes affected the quality of cortical bone. Bone is a dynamic organ that can change its shape and quality through bone formation and resorption to adapt to the mechanical environment, a process known as bone remodeling (Wolff, 1892). Osteocytes are mechanosensors of bone and play an important role in bone remodeling (Tresguerres et al., 2020). When external force is exerted on femur, the mechanical stimuli are transmitted from the organ scale to the cell scale step by step and ultimately sensed by osteocytes, which then release biochemical signals to activate bone remodeling (Klein-Nulend et al., 2013; Robling and Bonewald, 2020; Tresguerres et al., 2020). Finally, the shape and quality of the bone are changed to adapt to the mechanical environment. Therefore, the mechanical response of osteocytes plays an important role in regulating cortical bone quality. Organ, tissue, tissue element and cell four scales FE models of bone were developed that could investigate the mechanical responses of cortical bone at any regions of interest in our previous study (Cen et al., 2021). In addition, cortical bone loss in the mid-femoral neck occurred in elderly individuals with aging, and it showed heterogeneous changes in different regions (Looker et al., 1998). A quantitative analysis of the structural changes of the femoral neck in 100 women aged 20–90 years showed that cortical bone in the inferior (Inf) quadrant of the femoral neck was relatively thicker and had higher BMD, but it was thinner and had lower BMD in the other three quadrants of the femoral neck (Poole et al., 2010). The differences in cortical bone thickness and BMD among different quadrants of femoral neck caused the mechanical responses to be more complex in the elderly. Now, based on the quadrantal differences in bone quality of the mid-femoral neck, multiscale FE models of bone were developed to investigate the mechanical responses of the mid-femoral neck. The mechanical response analysis of cortical bone is crucial for the prediction of femoral neck strength and the assessment of FNF risk. The mechanical response of osteocytes plays a key role in understanding the regulatory mechanisms of bone remodeling. The comparison of mechanical response between cortical bone and osteocytes was the basis for exploring mechanosensation and mechanotransmission among different scales. However, the mechanical response analysis of femoral neck at the cell scale is scarce, especially in the context of the aging bone.

In this study, multiscale finite element (FE) models of the proximal femur in young and elderly people were developed. The mechanical responses at the tissue and cell scales in the mid-femoral neck, as well as the comparison of mechanical responses between the two scales were investigated. The results have important implications for exploring the relationship of bone quality and the mechanical responses among different scales and provide new insights for the pathogenesis for age-related bone loss.

## Materials and Methods

**FIGURE 1:**
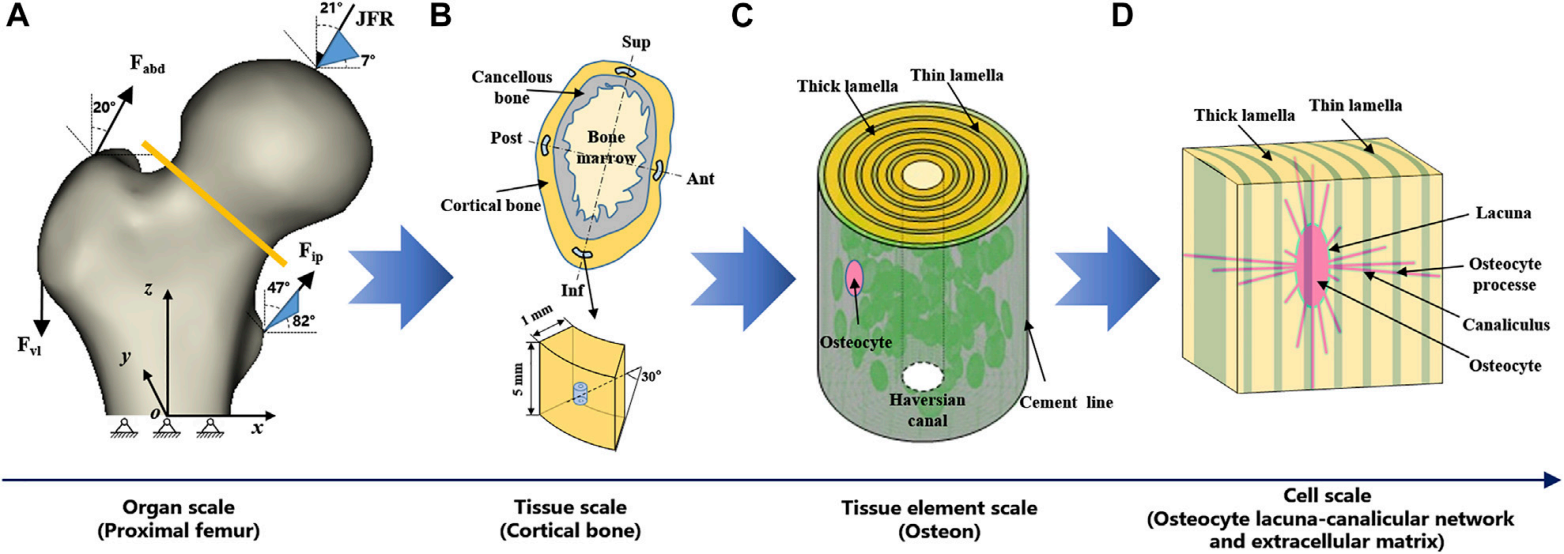
Multiscale FE models of proximal femur. **(A)** organ scale (proximal femur), **(B)** tissue scale (cortical bone), **(C)** tissue element scale (osteon), and **(D)** cell scale (OLCEM). Superior (Sup), Inferior (Inf), Anterior (Ant), Posterior (Post).

In this study, multiscale FE models of the proximal femur for young and elderly individuals were developed based on our previous study (Cen et al., 2021). The models included four scales, i.e., organ scale (proximal femur), tissue scale (cortical bone), tissue element scale (osteon), and cell scale (OLCEM), as shown in Figure 1. Differences in morphological and mechanical properties of the proximal femur at different scales between the elderly and young people were considered in the modeling process, especially in the mid-femoral neck region. Finally, the mid-femoral neck region was divided into four quadrants, i.e., superior (Sup), Inf, anterior (Ant), and posterior (Post). FE models of cortical bone, osteon, and OLCEM were located in the four quadrants of the mid-femoral neck. A similar method of modeling process has been described in our previous study (Cen et al., 2021). Now, a simple modeling process and necessary parameters of the multiscale FE models in this study were introduced here.

### Organ Scale FE Model: Proximal Femur

Proximal femur models of the elderly and young were established based on quantitative computed tomography (QCT) data. The models were a quarter of the full femur length for both the young and the elderly, as shown in Figure 1A. The models were meshed using Hypermesh 13.0 (Altair Engineering Corp., United States). The element sizes of the models at different scales were determined through a convergence analysis by gradually increasing the size of the elements until the deviations in the estimated strain reached <3%. The detailed element parameters are listed in Table 1. The BMD of each element in the proximal femur model was determined based on QCT data, as shown in Eqs 1, 2 (Schileo et al., 2008; Cen et al., 2021). The mechanical properties of the models were defined as orthotropic, and 121 discrete material sets were grouped based on the relationship between BMD and elastic constants, as shown in Table 2 (Enns-Bray et al., 2014). The loading condition of the mid-stance with single limb support in the walking gait was simulated. Three muscle groups (abductors, vastus lateralis, and iliopsoas) and the joint contact force (Figure 1A) were considered (Simões et al., 2000; Sangeux, 2019). The distal end of the proximal femur was rigidly constrained. The muscle forces were the average values for adult reported in the literatures (Simões et al., 2000; Diffo Kaze et al., 2017; Sangeux, 2019), and the muscle forces in the elderly were assumed to be 60% of those in the young due to age-related loss of muscle mass and strength (Doherty, 2001; Williams et al., 2002). The joint contact force was assumed to be twice the weight in the mid-stance of gait (Diffo Kaze et al., 2017). The values of the muscle and joint contact forces are listed in Table 3. Finally, the FE models were solved using Abaqus 6.14 (Simulia Corp., United States) for standard static analysis.

$$\rho_{\text{QCT}}\;=\text{ 0}\text{.}0008102\text{HU }+\text{ 0}\text{.}0026371\;\left({\text{g/cm}}^{3}\right)$$ (1)

$$\rho_{\text{ash}}\;=\text{ 0}\text{.}87719\rho_{\text{QCT}}\;+\text{ 0}\text{.}07895\;\left({\text{g/cm}}^{3}\right)$$ (2)

**TABLE 1:** Detailed element parameters of multiscale FE models.

FEA models	Models types	Number of nodes	Number of elements
Proximal femur	Young	38,148	2,04,622
	Elderly	31,543	1,67,827
Cortical bone	Young	21,033	17,920
	Elderly	21,033	17,920
Osteon	Young	7,88,690	44,76,206
	Elderly	4,23,458	23,53,219
OLCEM	Young	7,30,898	33,41,193
	Elderly	5,40,038	23,25,931

**TABLE 2:** Density–elasticity relationships used to determine the orthotropic elastic constants as functions of element ash density *ρ*_ash_ (g/cm^3^) (Enns-Bray et al., 2014).

Elastic constant (MPa)	Cancellous bone	Cortical bone
*E*_*x*_	*E*_z_ × 0.47 *ρ* ^0.12^	0.57 × *E*_z_
*E*_*y*_	*E*_z_ × 0.76 *ρ* ^0.09^	0.57 × *E*_z_
*E*_*z*_	10500 *ρ* ^2.29^	10500 *ρ* ^2.29^
*G*_*xy*_	*E*_z_ × 0.26 *ρ* ^0.24^	0.29 × *E*_z_
*G*_*yz*_	*E*_z_ × 0.29 *ρ* ^0.17^	0.2 × *E*_z_
*G*_*zx*_	*E*_z_ × 0.45 *ρ* ^0.18^	0.2 × *E*_z_
*ν*_*xy*_	0.27 *ρ* ^*−*0.09^	0.37
*ν*_*yz*_	0.14 *ρ* ^*−*0.16^	0.3
*ν*_*zx*_	0.14 *ρ* ^*−*0.07^	0.3

Note: The subscripts *x*, *y*, and *z* represent the directions along the normal directions of the sagittal, coronal, and transverse planes, respectively.

**TABLE 3:** Muscle and joint contact forces in the proximal femur models for the young and the elderly (Simões et al., 2000; Doherty, 2001; Williams et al., 2002; Diffo Kaze et al., 2017; Sangeux, 2019).

Load	Resultant force (N)		Angle (degree)	
	Young	Elderly	α	β
Joint contact force	1,100	1,170	21	7
Abductors	300	180	20	180
Iliopsoas	188	112.8	47	82
Vastus lateralis	292	175.2	180	—

Note: α represents the angle between the load direction and *z*-axis; β represents the angle between the load direction and *x* axis.

### Tissue Scale FE Model: Cortical Bone

Cortical bone models were segmented into four quadrants of the mid-femoral neck based on QCT data. The models were simplified as an arc shell of 30°, 5 mm length, and 1 mm thickness for both the young and the elderly, as shown in Figure 1B. The models were meshed with a denser mesh using Hypermesh 13.0, and the detailed element parameters are listed in Table 1. The models were defined as orthotropic mechanical properties and grouped into 37–68 discrete material sets. The boundary conditions of the cortical bone models were applied using the sub-model method, in which an interpolation of the calculated nodal displacements from the proximal femur models was applied to the peripheral nodes of the cortical bone models. The FE models were solved using Abaqus 6.14 for standard static analysis.

### Tissue Element Scale FE Model: Osteon

The osteon models were simplified as hollow cylinders, as shown in Figure 1C. The hollow space is the Haversian canal, which is surrounded by concentric lamellae. The outermost layer is the cement line. Each layer of lamellae contained five sub-lamellar layers and was divided into thick and thin lamellae because the orientations of the collagen fibers were different (Weiner et al., 1999). The lacunae were removed from the lamellae using MATLAB software (MathWorks, Natick, MA, United States). The differences in osteon morphology between the elderly and the young were considered in the modeling process, and the geometrical parameters of the osteon models are listed in Table 4, including the diameters of the osteon and the Haversian canal, thickness of thick and thin lamellae, and the sizes and densities of lacunae (Ardizzoni, 2001; Bernhard et al., 2013; Hemmatian et al., 2018). The models were meshed using Hypermesh 13.0, and the detailed element parameters are listed in Table 1. Numerical homogenization method was used to determine the mechanical properties of the thick and thin lamellae (Vercher et al., 2014; Tian et al., 2019). Differences in mineral content among the four quadrants of the mid-femoral neck in the elderly and young models were considered in the modeling process. That is, the mineral content of lamellae in the Inf quadrant of the mid-femoral neck was the highest and gradually decreased in the Post, Ant, and Sup quadrants. Meanwhile, the mineral content decreased in the four quadrants with aging (Looker et al., 1998; Poole et al., 2010; Bala et al., 2015). Therefore, the mineral contents of lamellae in the osteon models in the Sup, Inf, Ant, and Post quadrants of the mid-femoral neck of the elderly and the young were assumed to be 30%, 34%, 38%, and 42% vs. 36%, 40%, 42%, and 44%, respectively (Looker et al., 1998; Poole et al., 2010). In short, representative volume elements (RVEs) of the mineralized collagen fibrils were developed to determine the stiffness matrices of mineralized collagen fibrils based on the different mineral contents of the four quadrants of the mid-femoral neck in the elderly and young models. Then, the stiffness matrices of the five sub-lamellar layers were calculated through the Lekhnitskii transformation according to the orientations of the collagen fibers. Finally, the stiffness matrices of the thick and thin lamellae with different mineral contents were determined, and are listed in Table 5. The detailed process was described in our previous study (Cen et al., 2021). Note that the porosity of the lamellae was no longer introduced as an independent factor affecting the stiffness matrix, but as an influential factor on the mineral content of the lamellae. The material properties of the cement lines were assumed to be isotropic elastic for the young and the elderly models with E = 8 MPa and *ν* = 0.30 (Beno et al., 2006). The boundary condition of the osteon models was applied using the sub-model method, in which an interpolation of the calculated nodal displacements of the FE model of the cortical bone was applied to the peripheral nodes of the FE model of the osteon. The FE models were solved using Abaqus 6.14 for standard static analysis.

**TABLE 4:** The geometrical parameters of the osteon (Ardizzoni, 2001; Bernhard et al., 2013; Hemmatian et al., 2018).

Parameters	Young (μm)		Elderly (μm)	
Diameter of osteon	239	—	195.6	—
Diameter of Haversian canal	28	—	30	—
Layers of lamellae	—	30	—	24
Thickness of thick lamellae	4.5	—	4	—
Thickness of thin lamellae	1.4	—	1.4	—
Thickness of cement line	3	—	3	—
Number of lacunae	—	202	—	101

**TABLE 5:** The stiffness matrices of thick and thin lamellae with different mineral contents.

Mineral content (%)	Stiffness matrices of thin lamellae (MPa)	Stiffness matrices of thick lamellae (MPa)
30	$\left[\begin{array}{rrrrrr} 27052 & 9908 & 4012 & -753 & 824 & -124\\ & 23575 & 3796 & -753 & 203 & -293\\ & & 9692 & -94 & 1 & 0\\ & & & 5736 & -143 & 251\\ & symm & & & 4085 & 1040\\ & & & & & 6488 \end{array}\right]$	$\left[\begin{array}{rrrrrr} 11547 & 5269 & 3575 & 425 & -2157 & -93\\ & 29693 & 6539 & 1031 & -684 & -272\\ & & 23746 & 271 & -1482 & -134\\ & & & 3776 & 78 & 24\\ & symm & & & 7053 & -661\\ & & & & & 3148 \end{array}\right]$
34	[3059011089 4193−970889−12426109 3954−970210−32210293−10413−56357−154284symm443611917188]	$\left[\begin{array}{rrrrrr} 12411 & 5690 & 3700 & 501 & -2449 & 109\\ & 33786 & 7181 & 1298 & -811 & -291\\ & & 26125 & 329 & -1646 & -157\\ & & & 4133 & 86 & 27\\ & symm & & & 7819 & -767\\ & & & & & 3364 \end{array}\right]$
36	[3241011693 4308−1093906−130273534043−1093216−32510595−115−316669−158284symm461412577516]	$\left[\begin{array}{rrrrrr} 12850 & 5926 & 3774 & 567 & -2581 & 110\\ & 35895 & 7518 & 1443 & -872 & -298\\ & & 27256 & 362 & -1726 & -153\\ & & & 4320 & 89 & 31\\ & symm & & & 8183 & -796\\ & & & & & 3470 \end{array}\right]$
38	[34308123164407−1220 922−141286734134−1220 227−32510973 −118 −24107008−162278symm480013227852]	$\left[\begin{array}{rrrrrr} 13351 & 6150 & 3866 & -635 & -2704 & -106\\ & 38106 & 7865 & -1594 & -941 & 306\\ & & 28452 & -394 & -1816 & 143\\ & & & 4526 & 90 & 32\\ & symm & & & 8565 & 813\\ & & & & & 3592 \end{array}\right]$
40	[3604812885 4512−1328 927−14629915 4229−1328 233−32411353 −123 −28117328−163273symm497413778154]	$\left[\begin{array}{rrrrrr} 13832 & 6370 & 3967 & 685 & -2809 & 102\\ & 40107 & 8183 & 1736 & -1001 & -309\\ & & 29589 & 421 & -1910 & -138\\ & & & 4724 & 90 & 33\\ & symm & & & 8913 & -833\\ & & & & & 3714 \end{array}\right]$
42	[3771313435 4657−1419 943−15131157 4344−1419 239−32911751 −136 −2510 7644−165275symm515814318463]	$\left[\begin{array}{rrrrrr} 14330 & 6622 & 4078 & 726 & -2914 & 101\\ & 42010 & 8499 & 1856 & -1046 & -315\\ & & 30753 & 444 & -2006 & -139\\ & & & 4922 & 92 & 35\\ & symm & & & 9264 & -860\\ & & & & & 3843 \end{array}\right]$
44	[4045014376 4834−15221008−16133419 4479−1522 257−35212187 −154−15 6 8160−176295symm543615559028]	$\left[\begin{array}{rrrrrr} 14992 & 6977 & 4200 & 785 & -3172 & 106\\ & 45090 & 8991 & 2032 & -1130 & -336\\ & & 33014 & 481 & -2226 & -150\\ & & & 5182 & 94 & 38\\ & symm & & & 9916 & -935\\ & & & & & 4006 \end{array}\right]$

### Cell Scale FE Model: OLCEM

The OLCEM models were simplified as cubes for both the young and the elderly, as shown in Figure 1D. Each model contained osteocytes, perilacunar matrix (PCM), canaliculi, and ECM. The differences in OLCEM morphology between the elderly and young were considered in the modeling process, such as the sizes of osteocytes and PCM, and numbers of canaliculi and osteocyte processes (Ascenzi et al., 2004; Fratzl et al., 2004; You et al., 2004; Beno et al., 2006; Milovanovic et al., 2013; Buenzli and Sims, 2015). The geometrical parameters of the OLCEM models are listed in Table 6. The models were meshed using Hypermesh 13.0, and the detailed element parameters are listed in Table 1. The mechanical properties of the PCM and osteocyte were assumed to be isotropic elastic for the young and the elderly models with E = 40.00 kPa, ν = 0.40 and E = 4.47 kPa, ν = 0.30, respectively (Wang et al., 2015). The mechanical properties of thick and thin lamellae in the ECM were the same as those of the lamellae in the osteon models. The submodel boundary condition was also applied to the FE models of the OLCEM by interpolation from the osteon models. The FE models were solved using Abaqus 6.14 for standard static analysis.

**TABLE 6:** The geometrical parameters of the OLCEM models (Ascenzi et al., 2004; Fratzl et al., 2004; You et al., 2004; Beno et al., 2006; Milovanovic et al., 2013; Buenzli and Sims, 2015).

Parameters	Young (μm)	—	Elderly (μm)	—
Semiaxis of lacunae	3.465/7.305/10.405	—	2.145/4.505/5.865	—
Thickness of PCM	0.75	—	0.75	—
Number of canaliculi and osteocyte processes	—	18	—	14
Diameter of canaliculi and osteocyte processes	0.259/0.104	—	0.259/0.104	—
Length of ECM	45	—	45	—

## Results

In this study, multiscale FE models of the proximal femur in young and elderly people were developed. The mechanical responses at the tissue and cell scales in the four quadrants of the mid-femoral neck and the differences in mechanical responses between the two scales were studied. The minimum principal strain and mean SED were analyzed, since the minimum principal strain is one of the most effective way for osteocytes to sense mechanical stimulation (Ascenzi et al., 2013; Vaughan et al., 2013) and the mean strain energy density (SED) is considered to be closely related to bone formation (Schulte et al., 2013).

### The Minimum Principal Strain and SED Distributions at Tissue Scale

**FIGURE 2:**
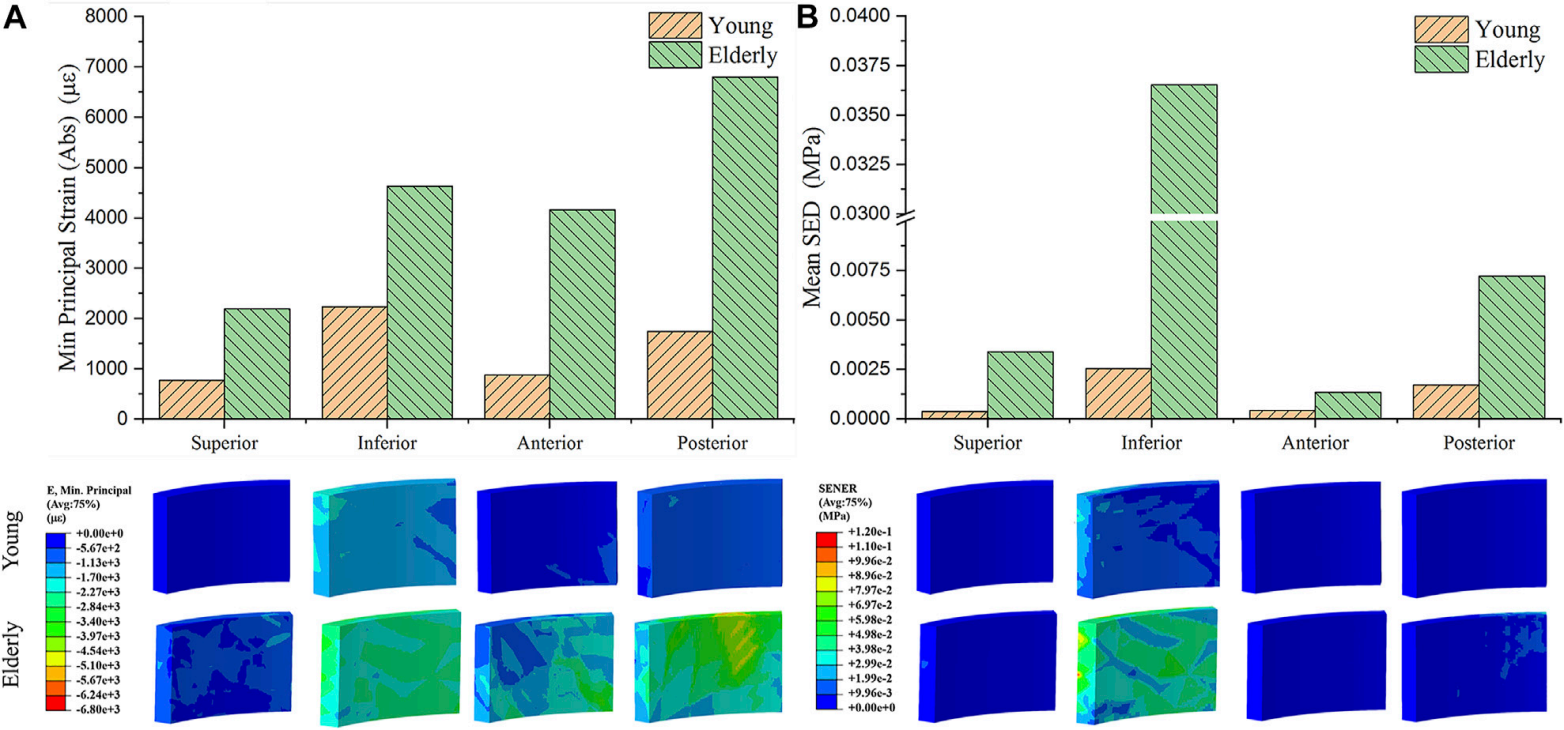
The minimum principal strain **(A)** and SED **(B)** of cortical bone in four quadrants of the mid femoral neck between the young and the elderly models.

Figure 2 shows the minimum principal strains and SEDs of the cortical bone in four quadrants of the mid-femoral neck in the young and the elderly models. The results showed that the absolute value of the minimum principal strains and mean SEDs of the cortical bone in the elderly were higher than those in the young. Furthermore, the differences in the minimum principal strain and the mean SED of the cortical bone among the four quadrants in the elderly were larger than those in the young. Specifically, the minimum principal strain of the cortical bone in the Post quadrant of the mid-femoral neck was the highest and gradually decreased in the Inf, Ant, and Sup quadrants in the elderly. The highest minimum principal strain of the cortical bone occurred in the Inf quadrant in the young model and gradually decreased in the Post, Ant, and Sup quadrants. Although the mean SEDs of the cortical bone in different quadrants of the mid-femoral neck in the elderly were higher than those in the young, their distribution trends among the four quadrants were consistent. The highest mean SED of cortical bone occurred in the Inf quadrant and gradually decreased in the Post, Sup, and Ant quadrants for both the elderly and young. Two ranges were calculated to quantitatively describe the differences in minimum principal strain and mean SED of cortical bone between the elderly and the young. The lower and upper limits of range were the values of minimum and maximum multiples that were calculated as the corresponding minimum principal strains (or the mean SEDs) in the elderly divided by that in the young, as shown in Table 7.

**TABLE 7:** The minimum principal strain and mean SED of cortical bone in the elderly and the young.

Absolute values of the minimum principal strain at tissue scale (με)				
Quadrants	Sup	Inf	Ant	Post
Elderly	2,193	4,633	4,163	6,803
Young	772.8	2,241	884.3	1,747
Multiples	2.838	2.067^a^	4.708^b^	3.894
**Mean SED at tissue scale (kPa)**				
**Quadrants**	**Sup**	**Inf**	**Ant**	**Post**
Elderly	3.39	36.54	1.33	7.21
Young	0.38	2.54	0.43	1.71
Multiples	8.921	14.386^b^	3.093^a^	4.216

a Represent the lower limits of range.

b Represent upper limits of range.

### The Minimum Principal Strain and SED Distributions at Cell Scale

**FIGURE 3:**
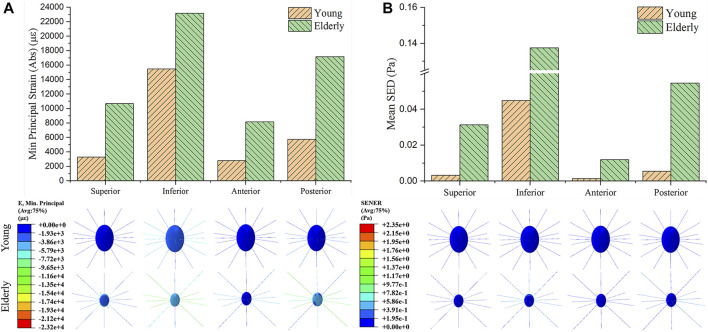
The minimum principal strain **(A)** and SED **(B)** of osteocyte in the four quadrants of the mid-femoral neck between the young and the elderly models.

Figure 3 shows the minimum principal strain and SED of the osteocyte in the four quadrants of the mid-femoral neck in the young and the elderly models. The minimum principal strain and mean SED showed the same distribution trends among the different quadrants of the mid-femoral neck for the young and the elderly. Specifically, the highest minimum principal strain and mean SED of osteocytes occurred in the Inf quadrant, and gradually decreased in the Post, Sup, and Ant quadrants. The absolute value of minimum principal strains and mean SEDs of osteocyte in the elderly were higher than those in the young. Two ranges were calculated to quantitatively describe the differences in minimum principal strain and mean SED of osteocyte between the elderly and the young. The lower and upper limits of range were the values of minimum and maximum multiples that were calculated as the corresponding minimum principal strains (or the mean SEDs) in the elderly divided by that in the young, as shown in Table 8.

**TABLE 8:** The minimum principal strain and mean SED of osteocytes in the elderly and the young.

Absolute values of the minimum principal strain at cell scale (με)				
Quadrants	Sup	Inf	Ant	Post
Elderly	10,670	23,160	8,155	17,180
Young	3,287	15,470	2,808	5,736
Multiples	3.246^b^	1.497^a^	2.904	2.995
**Mean SED at cell scale (Pa)**				
**Quadrants**	**Sup**	**Inf**	**Ant**	**Post**
Elderly	0.031	0.137	0.012	0.055
Young	0.003	0.045	0.001	0.005
Multiples	10.333	3.044^a^	12^b^	11

a Represent the lower limits of range.

b Represent upper limits of range.

### Comparison of Mechanical Responses Between the Tissue and Cell Scales

**FIGURE 4:**
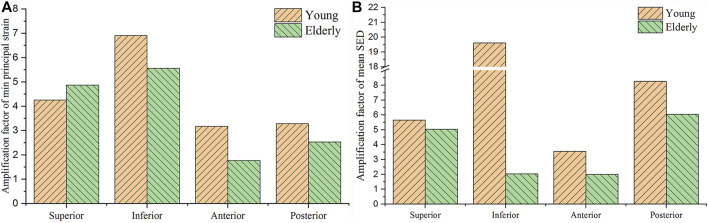
Comparisons of the amplification factors of the minimum principal strain **(A)** and the mean SED **(B)** in the four quadrants of the femoral neck in the young and the elderly.

To investigate the mechanotransmission between the tissue and cell scales, the strain and SED amplification factors were defined. The minimum principal strain amplification factor was calculated as the minimum principal strain of the osteocytes divided by the minimum principal strain of the cortical bone. The SED amplification factor was calculated as the SED of the ECM divided by that of the cortical bone. Figure 4 shows the minimum principal strain and SED amplification factors in the young and the elderly models. Because both the minimum principal strain and mean SED at the cell scale were higher than those at the tissue scale, the amplification factors were greater than one. The results revealed that both the amplification factors of minimum principal strain and mean SED showed large differences among the four quadrants of the mid-femoral neck. Most of the amplification factors in the young were higher than those in the elderly, except the amplification factor of minimum principal strain in the Sup quadrant. The amplification factor of minimum principal strain in the Sup quadrant in the young was 87.4% of that in the elderly. Specifically, the peak of the minimum principal strain amplification factor occurred in the Inf quadrant in both the young and the elderly models, and the values of the minimum principal strain amplification factor gradually decreased in the Sup, Post, and Ant quadrants. The mean SED amplification factor of the Inf quadrant was much greater than those of the other quadrants in the young models. The mean SED amplification factors were relatively low in the elderly models. Two ranges were calculated to quantitatively describe the differences in the amplification factors of minimum principal strain and mean SED between the elderly and the young. The lower and upper limits of range were the values of minimum and maximum multiples that were calculated as the corresponding amplification factor of minimum principal strains (or mean SEDs) in the young divided by that in the elderly, as shown in Table 9.

**TABLE 9:** The amplification factors of minimum principal strain and mean SED in the elderly and the young.

Amplification factor of the minimum principal strain				
Quadrants	Sup	Inf	Ant	Post
Elderly	4.865	5.563	1.76	2.525
Young	4.253	6.903	3.175	3.283
Multiples	0.874^c^	1.241^a^	1.804^b^	1.300
**Amplification factor of the mean SED**				
**Quadrants**	**Sup**	**Inf**	**Ant**	**Post**
Elderly	5.031	2.035	1.989	6.044
Young	5.656	19.612	3.548	8.261
Multiples	1.124^a^	9.637^b^	1.784	1.367

a Represent the lower limits of range.

b Represent upper limits of range.

c represents a special value which has been described specially in the main text Section (Section: Comparison of Mechanical Responses Between the Tissue and Cell Scales).

## Discussion

In this study, multiscale FE models of the proximal femur in young and elderly people were developed. The mechanical responses of cortical bone and osteocytes in the mid-femoral neck and the differences between the two scales were analyzed. The mechanical response analysis of cortical bone was crucial for the prediction of femoral neck strength and the assessment of FNF risk. The mechanical response of osteocytes was key to understanding the role of mechanical stimulation in bone remodeling and to exploring the regulatory mechanism of osteocytes in bone remodeling.

The strains and mean SEDs of the cortical bone models in the elderly were higher than those in the young, and significant differences in different quadrants were observed. The minimum principal strains in the Post and Inf quadrants of the mid-femoral neck was greater than those in the Ant and Sup quadrants, which is consistent with the results reported in the literature (Anderson and Madigan, 2013). The differences in mechanical responses among different quadrants were closely related to the anatomical structure, mechanical properties, and loading distribution of the proximal femur. The human hip joint is a ball-and-socket synovial joint formed by an articulation between the acetabulum and the femoral head. The acetabulum tilts forward, outward, and downward, respectively. In the mid-stance of gait, the body weight was transmitted to the femoral head and neck through the hip joint, and then to the femoral shaft. In addition, the muscles attached to the proximal femur produce some constraint forces. Finally, under combined loading, the cortical bone in the Sup quadrant of the mid-femoral neck bore tension. However, the cortical bone in the Inf quadrant is compressed. This was the main reason for the differences in the cortical bone mechanical responses among the different quadrants of the mid-femoral neck.

**FIGURE 5:**
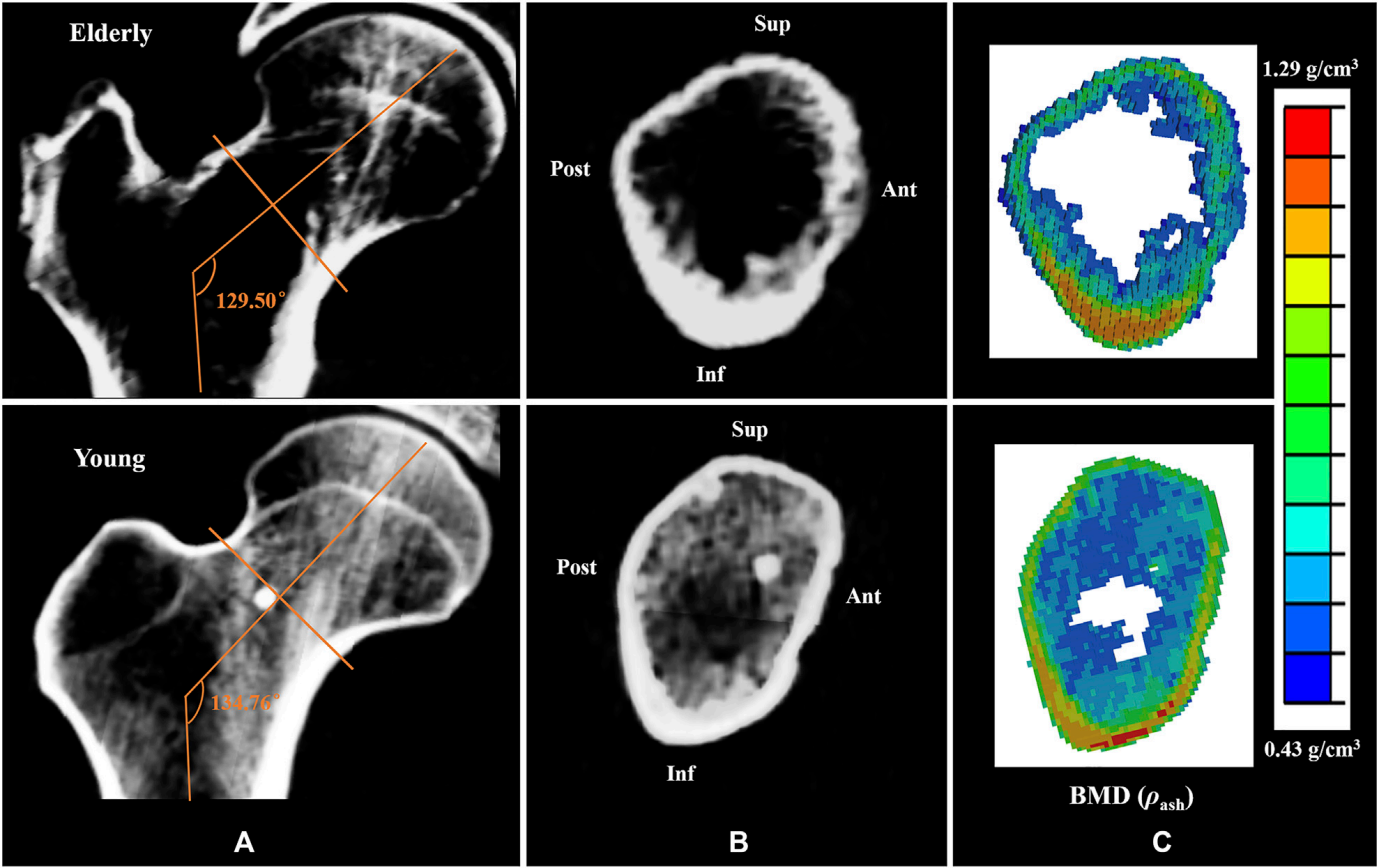
Comparisons of the CT scan slices and the BMD distributions between the elderly and the young. **(A,B)** the CT scan slices of the proximal femur and the mid-femoral neck, **(C)** the BMD distributions of the mid-femoral neck.

Generally, cortical bone in the Sup quadrant of the mid-femoral neck is thin and has low BMD. Cortical bone in the Inf quadrant is thick and has high BMD. With increasing age, loss of cortical bone in the Sup and Ant quadrants was more serious than that in the Inf and Post quadrants (Looker et al., 1998; Poole et al., 2010; Johannesdottir et al., 2011). Previous study has found that bone loss in the cortical bones of the mid-femoral neck in four different quadrants caused high strain and SED in the elderly models compared to those in the young models (Cen et al., 2021). This study found that the differences in bone mass and BMD among different quadrants in the elderly models aggravated the differences in cortical bone mechanical responses among different quadrants, which is consistent with the results reported in the literature (Bala et al., 2015; Cen et al., 2021). A numerical simulation study on the mechanical response of femoral neck showed that when the mechanical properties of the proximal femur in the young model were assigned to the mechanical properties of the elderly model, the strain increased by 42% (Anderson and Madigan, 2013). In this study, geometric models of the proximal femur were established based on QCT image data, and the mechanical properties of the proximal femur and cortical bone models were determined by the relationship between the gray value of QCT image data and elastic coefficients. Therefore, the morphologic and bone quality differences in different quadrants of the mid-femoral neck between the elderly and the young could be reflected very well in the FE models of the proximal femur and cortical bone, as shown in Figures 5A,B,C respectively. In addition, age-related loss of muscle mass and strength affects the mechanical response of cortical bone in the mid-femoral neck. Generally, human muscle mass and strength appeared to peak between the ages of 25 and 35, and decline annually, especially after the age of 60, with a rate of about 10% per decade (Doherty, 2001; Williams et al., 2002). One study showed that the constraint force of muscles attached to the proximal femur (abductor, vastus lateralis, and iliopsoas) further reduced the strain levels of the proximal femur (Simões et al., 2000). When the loading of the proximal femur in the young model was applied in the elderly model, the strain increased by 12% compared with that with its own loading (Anderson and Madigan, 2013). Therefore, the special morphology of the proximal femur and inhomogeneity of bone mass and BMD of the cortical bone caused differences in cortical bone mechanical responses among four quadrants of the mid-femoral neck. Furthermore, the inhomogeneous degradation of bone mass and BMD among four quadrants and age-related loss of muscle mass and strength in the elderly models aggravated the differences in mechanical response of cortical bone among quadrants. Those were also the main reason for the differences in the mechanical responses of cortical bone between the elderly and the young.

The mechanical responses at cell scale in the mid-femoral neck also showed quadrant differences. The mechanical response of osteocyte in the Post quadrant was no longer the maximum, but rather in the Inf quadrant. In fact, as mechanosensor of bone, osteocytes can sense mechanical stimuli to regulate bone remodeling, and cell strain is one way for osteocytes to sense mechanical stimuli directly (Klein-Nulend et al., 2013). The mechanical response of osteocytes affects cortical bone quality by regulating the bone remodeling process. An *in vitro* cell culture study showed that only high strain responses can generate biochemical responses in osteocytes (You et al., 2000). In this study, osteocytes in the Inf quadrant of the femoral neck experienced higher strain, and the cortical bone was thicker and had high BMD in the corresponding position. In contrast, osteocytes in the Ant and Sup quadrants of the femoral neck experienced lower strain, and the cortical bone was thinner and had low BMD, as shown in Figure 5B. This suggests that the strain response of osteocytes is closely related to cortical bone quality. Moreover, bone loss would decline under high strain environment, and the bone quality would be relatively better. In this study, high strain was mainly localized at the junctions of the osteocyte cell body and the processes, which are considered the most sensitive regions for osteocytes to sense mechanical stimulation (McNamara et al., 2009). Interestingly, integrins are attached to osteocyte processes. Therefore, osteocytes may regulate the bone remodeling process by adjusting the protein activity after sensing mechanical stimulation at the osteocyte processes.

Although the strain and mean SED of the proximal femur in the elderly models were higher than those in the young models at the tissue and cell scales, the minimum principal strain and mean SED amplification factors in the elderly models were lower than those in the young models. The minimum principal strain amplification factors of the elderly were lower than those in the young models by 19.4%–45.6%. These results indicate that the ability to sense strain stimuli for osteocytes in the elderly was weaker than that in the young. With increasing age, the densities of osteocytes and lacunae, and the number of canaliculi and osteocyte processes decreased (Milovanovic et al., 2013). Meanwhile, osteocytes became much smaller and rounder with aging (Hemmatian et al., 2018). These changes in osteocytes and LCNs were not conducive to sensing and transmitting mechanical stimuli in the elderly (Rath Bonivtch et al., 2007; Hemmatian et al., 2021), which was the main reason for the differences in the mechanical responses of osteocyte between the elderly and the young. Therefore, osteocytes require higher mechanical stimuli to regulate bone remodeling in the elderly. Our results showed that the minimum principal strain of osteocytes and the mean SED of ECM in the elderly were higher than those in the young, which is related to cortical bone loss in four different quadrants of the mid-femoral neck. This indicates that local cortical bone loss can enhance the mechanical response of osteocytes. Perhaps cortical bone quality could be improved by stimulating osteocytes. As a dynamic adaptive biomaterial, bone can change its shape and quality to adapt to the mechanical environment through bone remodeling. Generally, the elderly lack daily activities, and walking is their main activity. Although bone loss appeared at the femoral neck in the elderly, they could adapt to the mechanical environment of walking very well. However, it was difficult for the elderly femoral neck to bear high intensity loading. Therefore, sideways falls are the major cause of FNF in the elderly (Zani et al., 2015). Therefore, bone loss of femoral neck in the elderly was the result of adaptation to daily activities, and appropriate exercise may help improve the bone quality of femoral neck in the elderly.

In this study, a multiscale FE method was used to investigate the mechanical responses of proximal femur, and the differences in morphological and mechanical properties of the proximal femur at different scales between elderly and young people were considered in the modeling process. This study had some limitations. First, the proximal femur and cortical bone models were established based on CT image data from two participants, while only two participants were recruited in this study. Fortunately, the differences in bone morphology and bone quality among different quadrants of the mid-femoral neck between the elderly and young were in line with the characteristics described in the literature (Looker et al., 1998; Poole et al., 2010). Second, parameterized FE models at tissue element and cell scales were developed based on histological studies of human osteons and osteocytes instead of individual real morphologies of participants in this study. At present, it is difficult to acquire the actual morphology of living osteons and osteocytes. In addition, the parameterized model was more conducive to capturing morphological characteristics and comparing the effects of morphological differences on the mechanical responses. However, the assumption of an idealized geometry model may underestimate the strain compared with the real geometry model because the canaliculi and osteocyte processes with irregular shapes can generate stress and strain concentrations more easily than idealized straight cylindrical channels. Third, the validation of our models was limited by current experimental technologies that cannot directly measure the strain and SED of osteons and osteocytes *in vivo*. Fortunately, the strain distributions of the proximal femur and cortical bone models in the mid-femoral neck were in line with the results reported in the literature (Wik et al., 2010; Anderson and Madigan, 2013; Kersh et al., 2018); the strain response of osteocyte in this study was slightly lower than that measured by digital image correlation (DIC) technique (23,160 με vs. 35,000 με) (Nicolella et al., 2006), and was close to the results reported in numerical simulation studies (23,160 με vs. 6,600–26,000 με) (Rath Bonivtch et al., 2007; Verbruggen et al., 2012). New experimental platforms should be developed in future studies to verify the proposed model. Meanwhile, the FE models with the subject-specific joint and muscle forces as the boundary conditions are necessary to investigate the effect of boundary conditions on the mechanical responses in future study. Meanwhile, the sensitive analysis of the boundary conditions will be performed in future study.

## Conclusion

In this study, multiscale FE models of the proximal femur for young and elderly people were developed, and the mechanical responses of cortical bone and osteocytes in different quadrants of the mid-femoral neck were studied in the mid-stance of gait. The main conclusions are as follows:

1) The mechanical responses of cortical bone models showed significant differences in different quadrants of the mid-femoral neck, which was caused by the special anatomical morphology and inhomogeneous bone mass and BMD of the femoral neck.
2) The degradation of bone mass and BMD causes the minimum principal strains and mean SEDs in the elderly to be higher than those in the young. Inhomogeneous degradation of bone mass and BMD among four quadrants and age-related loss of muscle mass and strength in the elderly aggravated the differences in mechanical response of cortical bone among quadrants.
3) The higher the level of osteocyte mechanical response, the better the cortical bone quality in the mid-femoral neck.
4) The ability of osteocytes to sense strain stimuli in the elderly was weaker than that in the young. The cortical bone loss increased the mechanical response in the mid-femoral neck for the elderly, and enhanced mechanical stimulation of osteocytes.

## Data Availability

The original contributions presented in the study are included in the article/Supplementary Material, further inquiries can be directed to the corresponding author.
